# Analyzing post-COVID-19 demographic and mobility changes in Andalusia using mobile phone data

**DOI:** 10.1038/s41598-024-65843-2

**Published:** 2024-06-27

**Authors:** Joaquín Osorio Arjona

**Affiliations:** Department of Geography, Universidad Nacional de Educación a Distancia, 28040 Madrid, Spain

**Keywords:** Information technology, Scientific data

## Abstract

This work studies changes in the demographics of the different spatial units that make up the Andalusia region in Spain throughout the year 2021, with the aim of seeing the progressive recovery of the population after the COVID-19 pandemic. Mobile phone data from Origin–Destination matrices has been used, due to the ease of obtaining updated information quickly and constantly. A methodology has been developed to transform the number of travelers into an estimated population without biases, and an interpolation function has been used to take into account all the data available in the year 2021. Results show a direct link between the demographic changes in Andalusia and the removal of the mobility restrictions caused by the COVID-19 pandemic, with an increase of non-related work mobility and a decrease of static population. Travel distances between home and work places are also affected, with an increase of long trips after the end of the mobility restrictions. In addition, different patterns have been visualized, such as the concentration of commuting in the metropolitan areas of the region during working days, the population growth in rural areas during weekends, or the population displacement to coastal areas in summer.

## Introduction

Population data is the basic statistical information related to national economies and people’s livelihoods^1^. The human population of neighborhoods, cities or regions does not have a static number, but is a dynamic phenomenon that varies constantly both over on a daily and hourly basis and in different parts of a territory^2^. Mobility is an indispensable actor in population dynamicity, whose monitoring in space and time plays a fundamental role in a variety of fields such as demography or transport geography^3,4^. The major changes in the numbers, distributions and characteristics of population and mobility can occur in short periods, especially during natural disasters, conflicts or pandemics, such as the case of the health crisis caused by the COVID-19 virus^5–7^.

The simulation of mobility and population changes in space and time has become a crucial element in regional geography. Visualization of these changes is necessary to understand how the society occupying different parts of a territory functions and develop effective policies and programs^7–10^. Understanding the dynamic changes in human mobility and spatial interaction patterns at different spatial scales is crucial for analyzing the demographic impact of phenomena that strongly affect the territory, as is the case of the pandemic caused by the COVID-19 virus^11–14^. Therefore, spatial data with good quality, accurate, real-time, and high spatial granularity are needed to be able to accurately observe demographic changes according to different casuistries^15,16^.

However, as the population varies over time, census data are not adapted to dynamic models^17^. As both mobility and population undergo dramatic changes during a pandemic, static data such as those obtained from censuses and surveys become useless, as they are data that are not timely^18,19^ and are published annually or even over longer periods of time, making it very difficult to analyze and map changes in the territory over time periods shorter than one year^2,8,11,20^. Moreover, these static data only show the information of residents in their households. That means census databases are limited to the population at night, and not to the actual presence of people at different times of the day^9,13^. In addition, there are significant gaps in the quality and completeness of migration data^8^. This situation affects the production of population mapping, as it remains constrained by the logistics of the censuses and surveys^2^.

In the last two decades, the increasing use of data sources based on Information and Communication Technologies (ICT) have transformed the way in which the distribution of human population is studied and modeled in space and time^2,9,21^. Mobile phones are generating an enormous amount of data about human mobility even in low-income settings, and are having a great growth as demographic tools to observe the distribution of population in the territory over space and time^21–24^. Although mobile phone data usually show limitations in terms of accessibility, there is an increasing number of institutions that are betting on their enormous potential as sources of demographic information and that share this data openly and free of charge. This is the case of Spanish National Institute of Statistics (INE).

From this point of view of demography, mobile phone data offer the possibility of answering traditional questions about population while also suggesting new methods of asking questions^4^, since unlike census data, they can describe the behavioral patterns of a population in an up-to-date, dynamic, complete, and accurate way, in a short time and with low economic cost^6,19,25,26^. Mobile phones are an indispensable part of the daily lives of almost all sociodemographic groups in most of society, conferring them with a high penetration, which allows for the analysis of population distribution at a wide range of spatial scales, whether at the city or country level^2,9,27^. Other advantages include the high level of coverage over long periods of time^9^ and obtaining data from a large sample of users with greater coverage over the territory^28^.

The aim of this work is to study changes in mobility and population growth in different areas of the Andalusia region in Spain over a temporal period of 1 year, and to show the usefulness of mobile phone data for monitoring the evolution of demographics. The particular interest lies in the year of study, 2021. This year marked the end of the health crisis caused by COVID-19 and the progressive recovery of a situation of normality by society^29^. For this purpose, it is taken into account that the data correspond to a number of mobile phones detected, and a sample expansion methodology is proposed to visualize an estimated population in each flow of the Origin–Destination (OD) matrix. A way to analyze the evolution of the population of a region when the time scale consists of years (so there is no information regarding months or days) is to construct a synthetic population from known data^30^. This work proposes a methodology to build the population of the different units that make up the region of Andalusia from mobile phone data. Once the number of inhabitants of one day has been estimated, a dynamic and continuous population is built throughout the year using the INE population data only to define the number of inhabitants of the first day of the sample, while the number of inhabitants of the remaining days is based on an interpolation function that takes into account the estimated population of the other days in the sample.

This research has been supported by previous works that have carried out a methodology to estimate the population of a city at different times of a day, dividing the city into polygons based on the location of mobile phone antennas, mainly using the Voronoi method. This methodology has been applied in Paris^17^, Brno^31^, Helsinki^9^, Tallinn^22^, Beijing^32,33^, and Shanghai^34^. In contrast to these previous works, the time scale used is different. Instead of estimating the population of a territory over a day, this work estimates the population of a region over a full year. Deville et al.^2^ estimated population densities in periods of the year associated with normal mobility and in the summer period in France and Portugal with the objective of calculating the relative difference in density between those two periods and between the population density during a working day and a weekend. However, that work did not estimate the population continuously over a long period of time, but focused on specific periods of the year. Zu Erbach-Schoenberg et al.^21^ replicated this method to estimate seasonal changes in health district population of Namibia during a period of 3.5 years, generating static results on a monthly basis. In contrast, this research develops a methodology to estimate the population of the units that make up the study area on a constant basis over a full year and show them dynamically so the full-time scope can be studied in high detail. For that, a dynamic web map (which is available in the repository link written in the Data availability statement section) has also been elaborated.

Following this introduction, “Study area” will present the study area, “Data and methodology” will review the data used and the methodology employed in the research. Finally, in “Results”, the obtained results will be analyzed and a series of conclusions will be drawn in “Conclusions”.

## Study area

Andalusia, located in southern Spain, is the autonomous community with the largest population in the country (with a population of 8,500,187 inhabitants in 2022 according to the Municipal Register of Inhabitants prepared by the Institute of Statistics and Cartography of Andalusia). Andalusia has a unique geographical framework formed by Sierra Morena, the Betica mountain ranges, and the depression of the Guadalquivir Valley. This geographical disposition and the large territorial extension of the autonomous community have woven a wide network of cities along the coast and the inland countryside of the region. As a result, Andalusia has a high level of urbanization with approximately 30 cities with more than 50,000 inhabitants, concentrated mainly along the Guadalquivir Valley and on the coast. The most important cities in the region are Seville and Malaga with more than 500,000 inhabitants, and Cordoba and Granada with more than 200,000 inhabitants.

**Figure 1:**
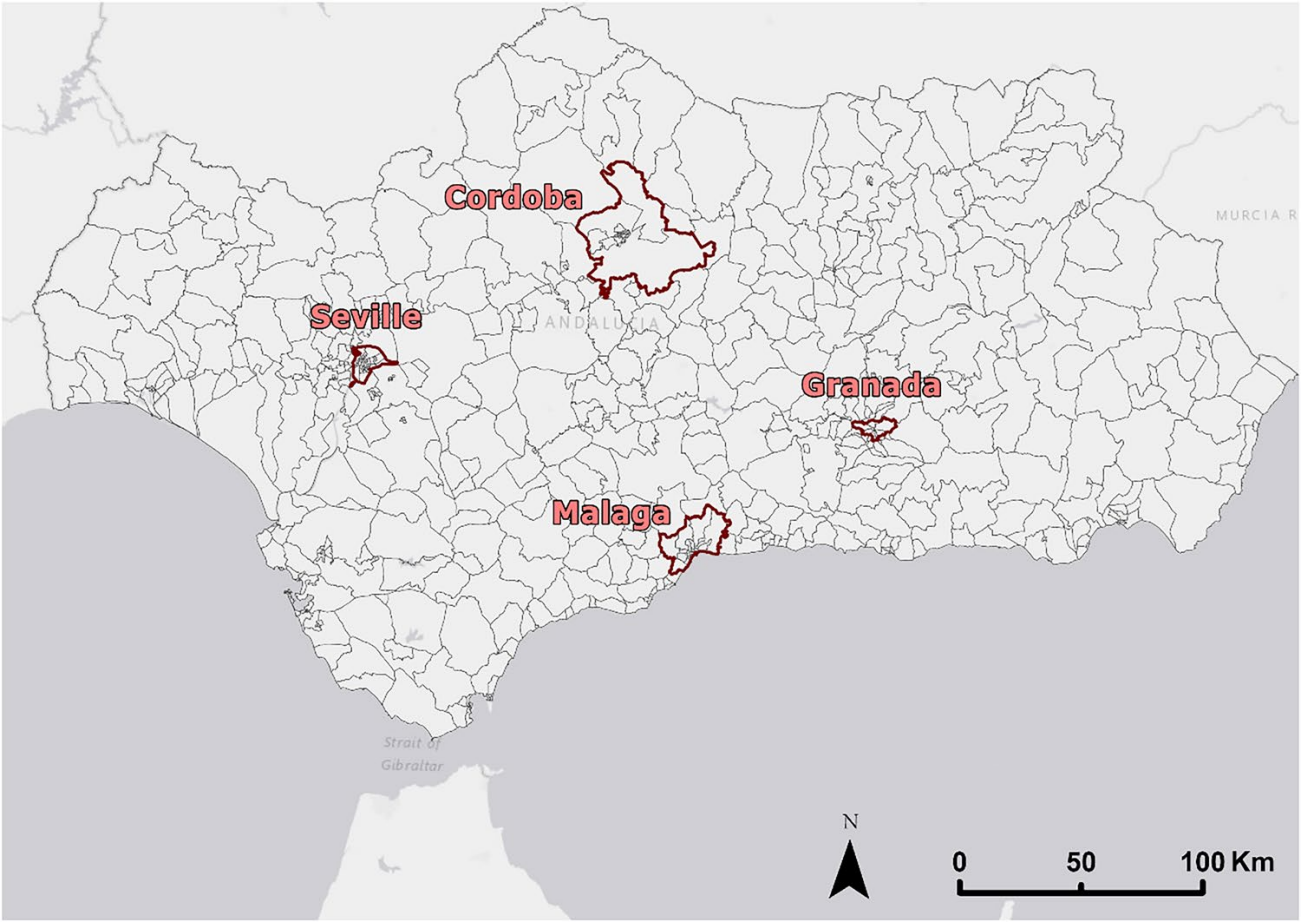
Population units for Andalusia designed by the INE. Source: Own elaboration based on INE data from the *Estadística Experimental* portal. The program used to create the figure is *ArcGIS Pro* 3.2.1 GIS software. The figure displays the 565 population units designed by INE. These units have a population referring to January 1, 2020, and have been designed to have a homogeneous population that exceeds the threshold of 5000 registered inhabitants and does not exceed a threshold of 50,000 inhabitants. For reference, the four major cities of the region (Seville, Malaga, Granada and Cordoba) have been signaled in the map.

The spatial scale used in this work consists on population cells or units supplied by INE in shapefile format. These vector units have been created so that each units have a population between a minimum of 5000 inhabitants and a maximum of 50,000 inhabitants. In this way, cities with a larger number of inhabitants are divided into districts or neighborhoods. By implementing these population units, the administrative and functional structure of the cities in the territory is maintained when estimating their population from mobile phone data. Andalusia has a total of 565 population units with population information referring to January 1, 2021 (Fig. 1).

## Data and methodology

The data used for this study were the daily mobility files downloaded from the INE's Experimental Statistics portal. These data are mobile phone records supplied by the three main telephone companies in Spain (*Movistar*, *Vodafone*, and *Orange*). Roaming users have already been filtered from the files. Each file corresponds to a day, being available only the files of Wednesdays and Sundays of each week from January to December 2021. Wednesdays reflect typical workday mobility patterns and tend to have some consistency with the mobility behaviors of other workdays. On the other hand, Sundays are generally considered rest days, which allows other mobility behaviors to be captured. It must be taken into account, however, that the availability of data only on these 2 days of the week may lead to the introduction of biases.

The file consists of the records of an OD matrix with a number of mobile phones that made a trip during a day, so each record corresponds to a trip flow. This record has a source unit identifier, a destination unit identifier, the date of the day, and the number of mobile phones detected in that flow. There are 819,898 Origin–Destination travel records published in 2021 whose origin and destination correspond to a unit of the autonomous community of Andalusia.

Then, the centroids from the 565 population units were generated. With these points, a layer of OD lines was created, with 319,225 relations. By joining the layer of lines of the OD matrix with the table of mobile phone records, a dynamic layer of OD matrices of trips was obtained, in which each record has a specific date. The matrix flows correspond to the number of mobile phones in the used tables, so they were expanded to show movement of an estimated total population as will be explained at the end of the section.

The next step was to sum the number of mobile phones available in each unit by both the source unit identifier and date and the destination unit identifier and date. As a result, two tables were obtained. The first table corresponds to the number of mobile phones that have left the unit of origin during a given day, and the second table is the number of mobile phones that have entered a destination unit during a given date. From these two tables, a single table of 58,758 records was created in which each row has the fields explained in Table 1.

**Table 1:** Fields of the table of mobile phone records by population unit and date. Source: Own elaboration based on INE data from the Estadística Experimental portal. Each record contains the code and name of the unit, an order value based on the date and used for the estimation and interpolation of the population, a correction factor based on the percentage at the provincial level of population with mobile phones and downloaded from the National Markets and Competition Commission website, a series of values ​​based on the number of mobile phones obtained, a series of values that show the obtained population from the calculations explained in the document, a smoothing factor value obtained from the mean and variance of all the population stocks values of the recording unit, and the estimated population value.

Field	Meaning	Example
Unit	Identifying code of the population unit	19MA
Unit name	Name of the population unit	Antequera
Date	Date on which the population unit is placed	12/01/2021
Order	Order value of the date over the year total per unit	3
Start-up population	Population of the unit corresponding to the beginning of the registration date (being the estimated population on the previous date, with the exception of the first registration date of the year, for which the population value corresponding to INE data from January 1, 2021 is used)	44,231
Correction factor	Percentage at the provincial level of the population whose mobile phone belongs to Movistar, Orange or Vodafone companies	77.42%
Stock of mobile phones	Number of mobile phones kept in the registration unit all day	26,723
Loss of mobile phones	Number of mobile phones with origin in the registration unit	2053
Gain of mobile phones	Number of mobile phones with destination in the registration unit	3407
Mobile phone balance	Subtraction between Gain of mobile phones and Loss of mobile phones	1354
Mobile phone population	Addition between mobile phones and Balance of mobile phones	28,077
Stock of population	Value obtained by applying the correction factor to the mobile phone population	36,266
Fixed population	Population of the unit corresponding to INE data as of January 1, 2021	43,831
Mean of the population unit	Average obtained from all the population stock values of the recording unit during the year	33,876.20
Variance of the population unit	Variance obtained from all the population stock values of the recording unit during the year	7,020,950.24
Smoothing factor	Correction value obtained from the square root of the variance over the fixed population value	12.65
Estimated population	Estimated population on the day from the population stock, using the formula described in the section	44,503

The population of mobile phones available in a population unit during a given date is determined from the sum of the stock of mobile phones (number of mobile phones that have remained in the unit) and the balance of mobile phones that have moved, this balance corresponding to the subtraction between the number of mobile phones that have entered the unit and the devices that have left the unit. However, studies using mobile phone data only account for a fraction of the total population^11,18,19^. Using the number of mobile phones to estimate demographic changes in a region leads to the introduction of a number of demographic and socioeconomic biases^18,33^. Therefore, the number of devices obtained was expanded using a correction factor. This factor corresponds to the percentage of the population that owns a mobile phone that corresponds to any of the three major telephone companies in Spain. This percentage is available at the province level, so the correction factor used to expand the number of mobile phones varies throughout the territory. Although the use of information on the population with mobile phone ownership helps to reduce as much as possible the biases obtained from using only mobile phone data and takes in account the population whose mobile phones operate under operators not included in the INE data, its effectiveness depends on the scale and precision of this data. It must be taken into account that this data is only available at the provincial level, so it does not have the greater accuracy that a municipal scale presents, so although the biases are mitigated, they may not have been completely eliminated.

The formula used for the calculation of the population stock $h$ of a population unit $c$ in a day $d$ was (1):

1 $${h}_{cd}=\frac{{m}_{cd}\times 100}{{s}_{p}}$$

where $m$ corresponds to the number of available mobile phones calculated in a unit $cd$, and $s$ is the expansion factor of a province $p$.

Although the calculated population stock is a value that estimates the population for 1 day, it is necessary to interpolate all the values obtained throughout the year so as to obtain a meaningful population evolution without the noises that can be caused by the impact of mobile phone use in certain temporal periods^17^. If this interpolation was not performed and the population stock was kept as the initial population on the following day, calculating the population in a linear way would lead to calculation errors that would result in continuous increases or decreases that would result in exaggerated values.

To estimate the population of each population unit on each day of the year 2021, all the population stock values calculated with the previous formula were used to obtain the mean and variance for each unit. Next, a smoothing value $s$ was established for each population unit $c$, using the official population data as of January 1 to adjust the interpolation and increase the accuracy of the final result^2^. For this purpose, the following formula was used (2):

2 $${s}_{c}=\sqrt{\frac{{v}_{hc}}{{i}_{c}}}$$

where $v$ is the variance of the population stock $h$ of a population unit $c$, and $i$ corresponds to the population of the unit $c$ according to INE data as of January 1, 2021.

Finally, the estimated population $p$ of a population unit $c$ in a day $d$ was calculated using the following formula (3):

3 $${p}_{cd}={i}_{c}+ \frac{{h}_{cd -} {n}_{hc}}{\sqrt{{s}_{c}}}$$

where $i$ is the population of the unit $c$ according to the INE data of January 1, 2021 used previously, ${h}_{cd}$ corresponds to the population stock $h$ of a population unit $c$ in a day $d$, $n$ is the average of the population stock $h$ of the unit $c$, and $s$ is the smoothing value of the unit $c$ calculated previously.

To evaluate the precision of the used data and the uncertainty of the results, an Ordinary Least Squares (OLS) Regression has been carried out between the official INE population data of the spatial units and the average obtained from all the population stock values of each spatial unit during the year. A Moran's I test for spatial autocorrelation has also been performed to analyze the spatial effect on the residuals.

Additionally, the dynamic layer of travel OD matrices was expanded by two different methods. The population $p$ corresponding to an external mobility flow $f$ from a day $d$ (i.e., flows whose origin and destination correspond to two different units) were expanded using the following formula (4):

4 $${p}_{fd}=\frac{{i}_{o}}{{m}_{fd}} \times 100$$

where $i$ corresponds to the population of the unit of origin $o$ according to INE data as of January 1, 2021, and $m$ is the number of mobile phones recorded in the flow $fd$. An internal mobility flow does not show variations in space, so in this case the population was calculated with the same formula used to expand the data from the table of mobile phone records by population unit and date (based on the correction factor that is equivalent to the percentage of mobile phones that the population of the unit corresponding to the internal flow has, varying this percentage according to the province of the unit).

The methodology carried out can be replicated in other geographical areas as long as OD matrices that contain data on the number of travelers in each flow are used and as long as information on the percentage of the population that owns a mobile device is available. Although this methodology can be replicated at any spatial scale, the results will be more reliable and precise the greater the spatial detail of the data.

## Results

The results for each moment of the entire year are available on the web map attached in the Data availability statement section. For the visualization of specific mobility patterns and demographic changes throughout the year 2021 in this section, eight days corresponding to four different periods of the year have been selected, so that the digital footprint obtained can be seen both on a working day and on a weekend day (Table 2).

**Table 2:** Relationship between the mapped day and the number of each of the maps that make up Figs. 2 and 4. Source: Own elaboration based on INE data. Four periods have been used to cartography the obtained results: COVID-19 state of alarm, end of state of alarm and return to normality, summer holidays, and period of normality. For each period, a work day coinciding with Wednesday has been selected, and a weekend day coinciding with Sunday, both days belonging to the same week in all cases. In total, eight days have been mapped.

Number	Day	Type	Period
1	January 20	Weekday	COVID-19 state of alarm
2	January 24	Weekend	COVID-19 state of alarm
3	May 19	Weekday	End of state of alarm and return to normality
4	May 23	Weekend	End of state of alarm and return to normality
5	August 18	Weekday	Summer holidays
6	August 22	Weekend	Summer holidays
7	November 24	Weekday	Period of normality
8	November 28	Weekend	Period of normality

**Figure 2:**
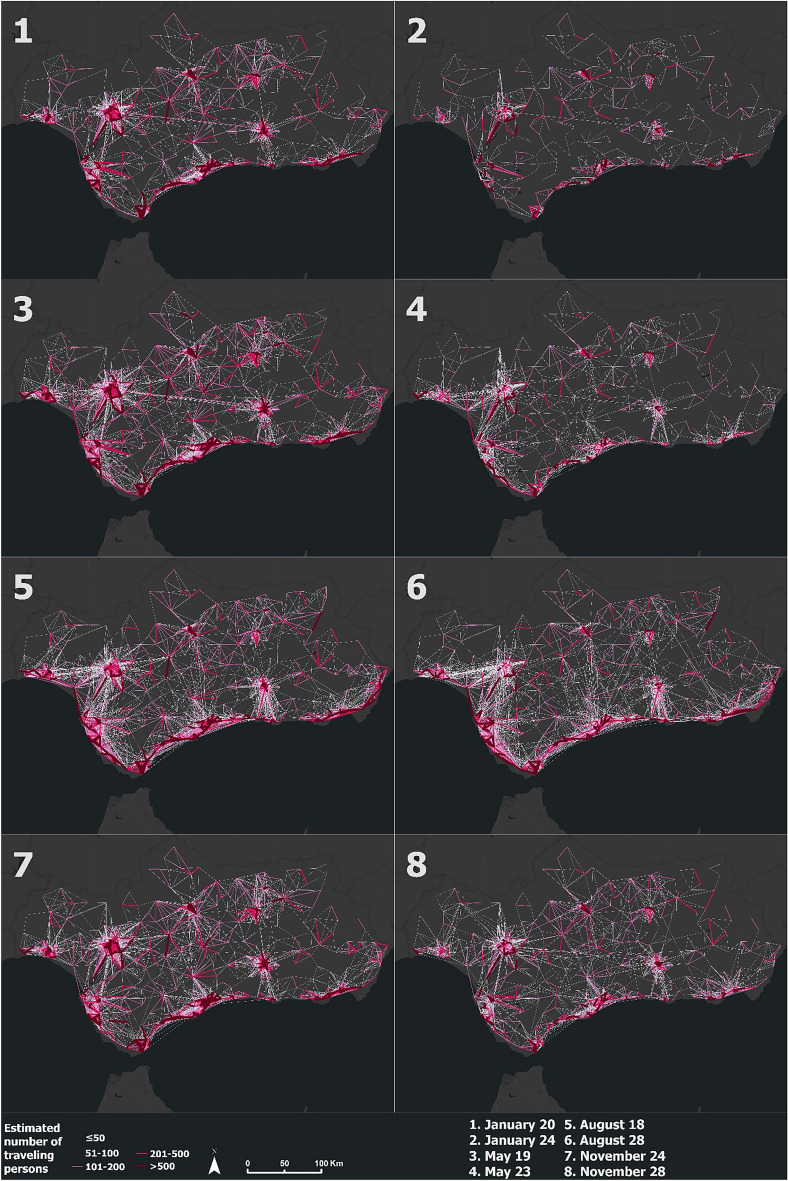
Origin–Destination population flows calculated from mobile phone data during the eight days defined in Table 2. Source: Own elaboration based on INE data from the *Estadística Experimental* portal. The program used to create the figure is *ArcGIS Pro* 3.2.1 GIS software. The figure shows an Origin–Destination matrix that indicates the number of an estimated population that have moved from a population unit to another one for the eight mentioned days.

Mobility in Andalusia over the year 2021 is mainly concentrated in the province capitals and along the Mediterranean coast. There are more trips during working days, and a reduction in mobility on weekends. This result indicates that the flows collected correspond mainly to trips from the home of residence to the place of work, and vice versa. It is also visualized as the period of the year with a lower number of trips and estimated population flows corresponds to the first months (Fig. 2, panels 1 and 2). This is due to the limited mobility caused by the state of alarm due to the COVID-19 pandemic that the region had until May 9. These restrictions included the perimeter closure of the municipalities for private vehicles and the limitation of the occupancy of public transport vehicles. Starting in May, there has been a progressive increase in mobility due to the end of mobility restriction policies and the rise in mobility caused by the vaccination campaign (Fig. 2, panels 3 and 4), culminating in summer, period with a large number of trips from the interior of the region to the coastal areas. At the same time, there has been a decrease in mobility in the main cities of the Guadalquivir Valley, situation caused due to very high temperatures and migration to the coast (Fig. 2, panels 5 and 6). In the last four months of the year (like November), the population has already recovered its daily activity to a casuistry similar to that before the COVID-19 outbreak in the year 2020 due to the recovery of mobility flows other than those that go from residence to work. At this period, the number of trips and population flows remains high and stable (Fig. 2, panels 7 and 8).

**Figure 3:**
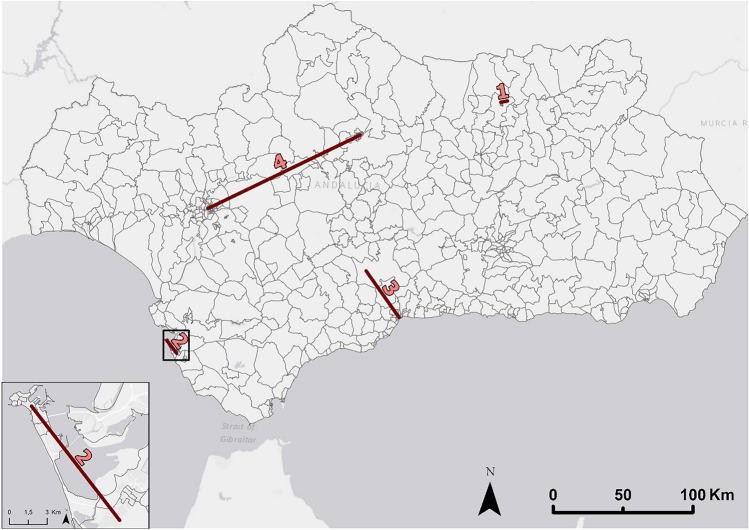
Origin–Destination flows whose estimated traveling population has been analyzed and visualized in Fig. 4. Source: Own elaboration based on INE data from the *Estadística Experimental* portal. The program used to create the figure is *ArcGIS Pro* 3.2.1 GIS software. The figure shows four Origin–Destination flows that represent determined study cases [(1) mobility between residential unit and industrial unit of the same municipality, (2) mobility between two municipalities of a metropolitan area, (3) mobility between two connected municipalities of a province, and (4) mobility between two tourist cities] further analyzed and visualized in Fig. 4.

Next, four mobility flows belonging to different cases are analyzed (Fig. 3). There is a direct link between the number of trips that take place between two cities and the distance between them. The OD flows with the highest number of passengers correspond to the lines that connect the population units of the same municipality. Meanwhile, the flows that involve larger trips and connect two different municipalities have a smaller number of travelers, this figure being smaller if the municipalities belong to different provinces, regardless of their population. Population is another important factor: if one or two of the municipalities connected by a flow that involves longer travel trips has a significant population weight, a flow can be visualized with a low number of travelers, but if the population of the two municipalities is not high, it is very possible that there will not be a flow of mobility. As will be seen, both the number and distance of trips and the population factor are affected by mobility restriction policies such as that caused by COVID-19 at the beginning of the year.

**Figure 4:**
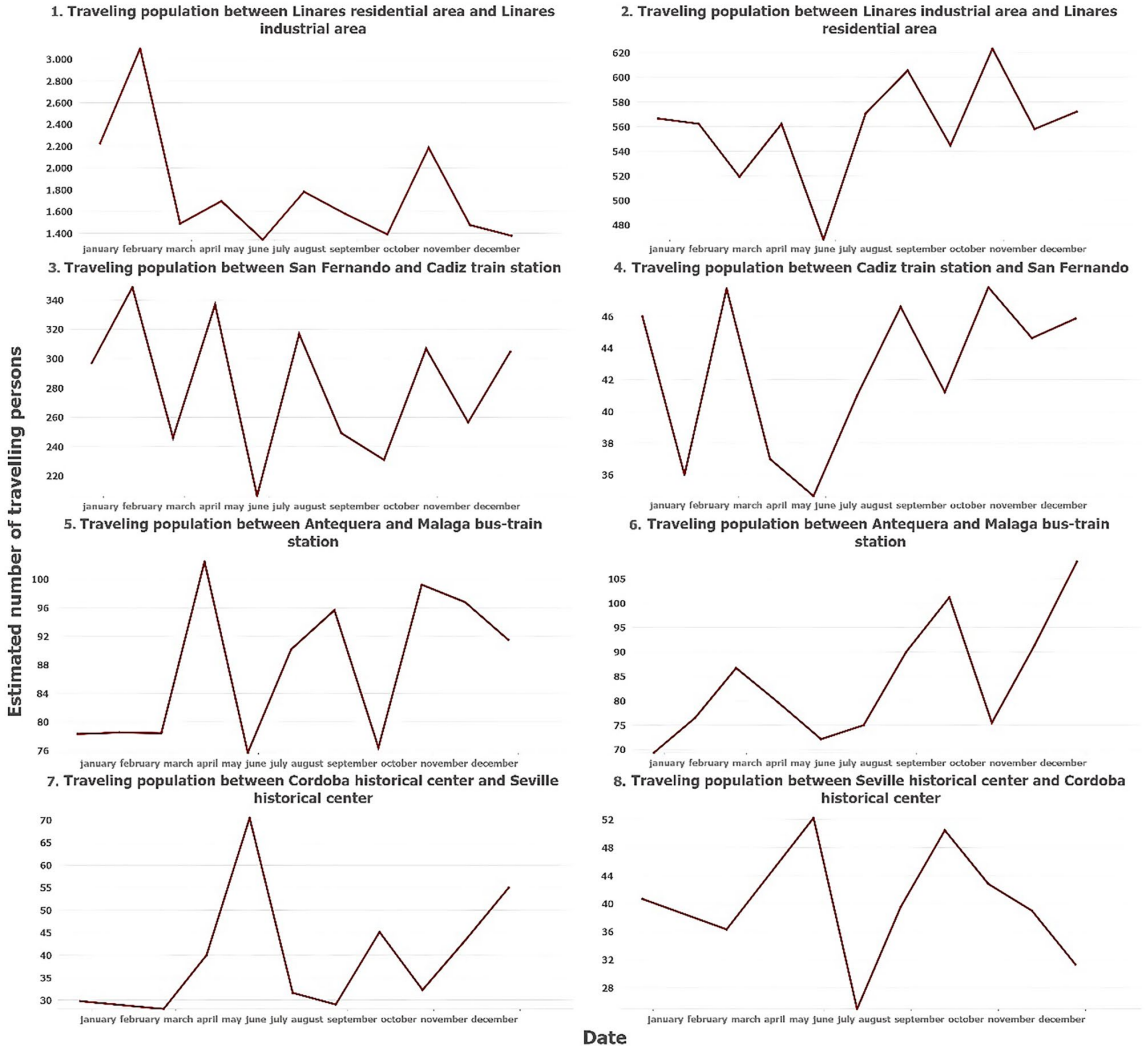
Examples of estimated number of travelling population in various Origin–Destination flows through the year 2021. Source: Own elaboration based on INE data from the *Estadística Experimental* portal. The program used to create the figure is *ArcGIS Pro* 3.2.1 GIS software. The figure shows graphs that indicate the estimated population that has moved from a particular population unit to another particular unit, in both directions. Four cases have been represented (mobility between residential unit and industrial unit of the same municipality, mobility between two municipalities of a metropolitan area, mobility between two connected municipalities of a province, and mobility between two tourist cities), so there is a total of eight graphs.

In the case of Linares (an average city in the interior of the Guadalquivir Valley), there is a large number of inhabitants traveling from residential neighborhoods to the industrial estate, especially at the beginning of the year (Fig. 4, panel 1). In contrast, mobility from the industrial estate to the place of residence is considerably lower, and is concentrated mainly in the second half of the year (Fig. 4, panel 2). This may indicate that commuting to work was the only type of mobility available during the COVID-19 mobility restriction policies, while with the end of the restriction measures, other types of mobility such as trips to shops or leisure centers have returned. The mobile phone data also shows population flows that travel from the dormitory towns to the central city of a metropolitan area, with considerably fewer travelers making the reverse route (as in the case of mobility between the dormitory town of San Fernando and the unit that houses the train station and the port of Cadiz, the main core of its metropolitan area). In both cases, mobility is stable in the first months of the year (except March), since even with mobility restriction policies due to COVID-19, public transport services are available that allow movement from residence to work. There is a decrease in mobility in the month of June, and a subsequent recovery in in autumn, where mobility flows are even greater than at the beginning of the year due to the return to a normal situation (Fig. 4, panels 3 and 4).

These behaviors are similar in the relationships between a medium-sized city and a provincial capital with a strong labor market (as in the case of the flow of trips between Antequera (average city located in the north of the province of Malaga) and the corresponding unit with the bus and train station in the city of Malaga), although the greater the distance of the trip, the fewer the number of travelers captured. Furthermore, the mobility restriction caused by COVID-19 significantly affects the reduction in mobility the greater the distance between two municipalities as can be seen in the relationship between the two spatial units in the first months of the year and the increase in the number of travelers starting in June with the end of mobility restrictions (Fig. 4, panels 5 and 6). Distance also explains the lower number of travelers estimated between the historic centers of two populated cities with strong tourism activity, as is the case of Seville and Cordoba. While the three previous examples showed a predominant mobility from home to work, in this case mobility is mainly of a tourist nature, with an almost identical number of travelers in both directions, with a reduced flow of travelers in the first months of the year and a greater number of people during the month of June, and in minor degree, the months of May and September. With the end of the mobility restriction measures in May, it can be seen that tourism began to recover precisely from this month and June (taking into account the pronounced decrease in tourist mobility in the summer months due to the climatic conditions of the region, which can reach temperatures above 45 °C). (Fig. 4, panels 7 and 8).

**Figure 5:**
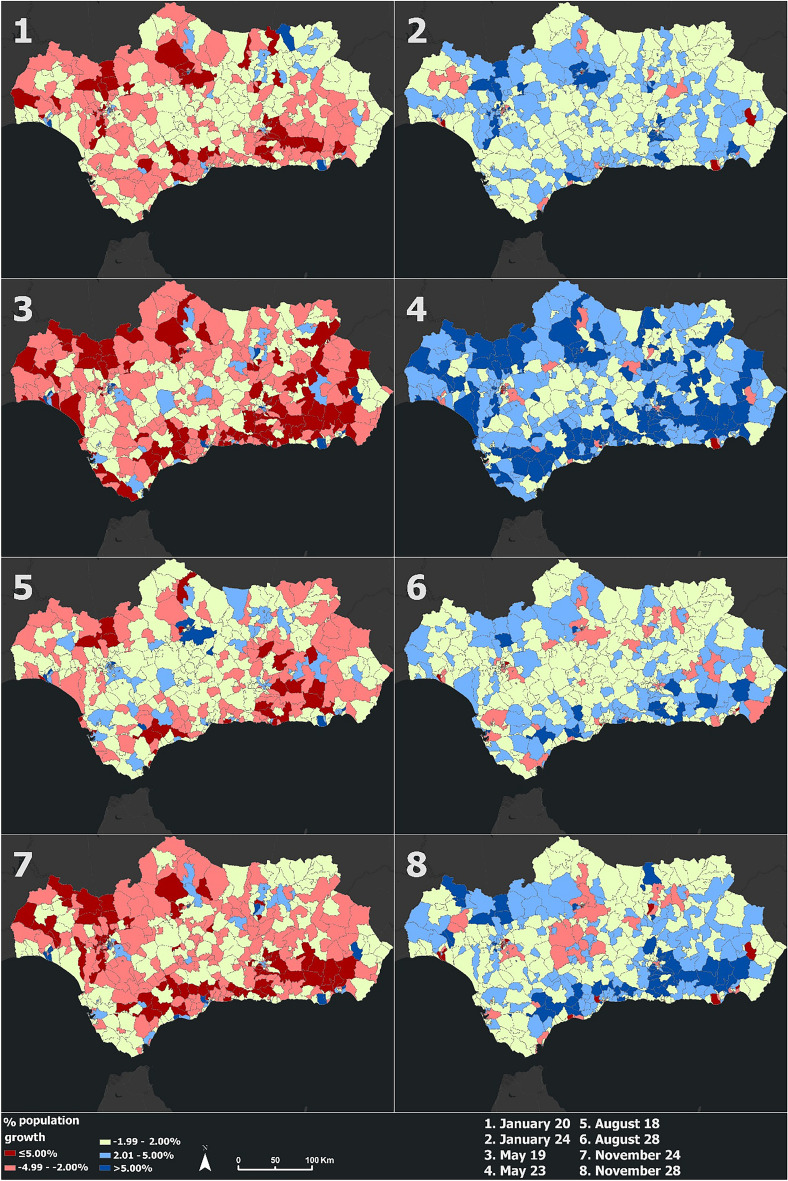
Population growth in Andalusia spatial units from mobile phone data during the eight days defined in Table 2. Source: Own elaboration based on INE data from the *Estadística Experimental* portal. The program used to create the figure is *ArcGIS Pro* 3.2.1 GIS software. The figure shows a choropleth representation of the percentage of population growth of each unit in Andalusia for the eight mentioned days.

Andalusia presents a generalized decrease in population in a large part of its territory (mainly in the towns located in the mountains) during working days, while the units corresponding to the historical centers and the work areas of the provincial capitals, industrial cities (Linares) or port cities (Malaga) show a demographic increase. This behavior is reversed on weekends, and can be linked to home-work mobility. In the first months of the year, demographic changes tended to be low or neutral, due to the low mobility and exits from the residential spatial units (Fig. 5, panels 1 and 2). However, there is a strong percentage of demographic change in the month of May compared to previous months, a situation that may be due to the end of the state of alarm caused by COVID-19 and the restoration of mobility in the region, which leads to higher percentages of variation. In addition, the month of May coincides with various cultural events of great importance in the region (such as the pilgrimage to the hermitage of El Rocío, located in the province of Huelva) (Fig. 5, panels 3 and 4).

During summer, population growth tends to be positive on weekdays and negative (especially in the mountain ranges in the east of the region) on weekends. This may be due to the strong tourism that the region experiences during this period, orienting labor mobility to this sector (Fig. 5, panels 5 and 6). Finally, in the season of autumn, we see greater changes in the percentage of population growth than during the first months of the year, this situation being due to the normalization of the daily life of the citizens and the recovery and increment of mobility in the region (Fig. 5, panels 7 and 8).

**Figure 6:**
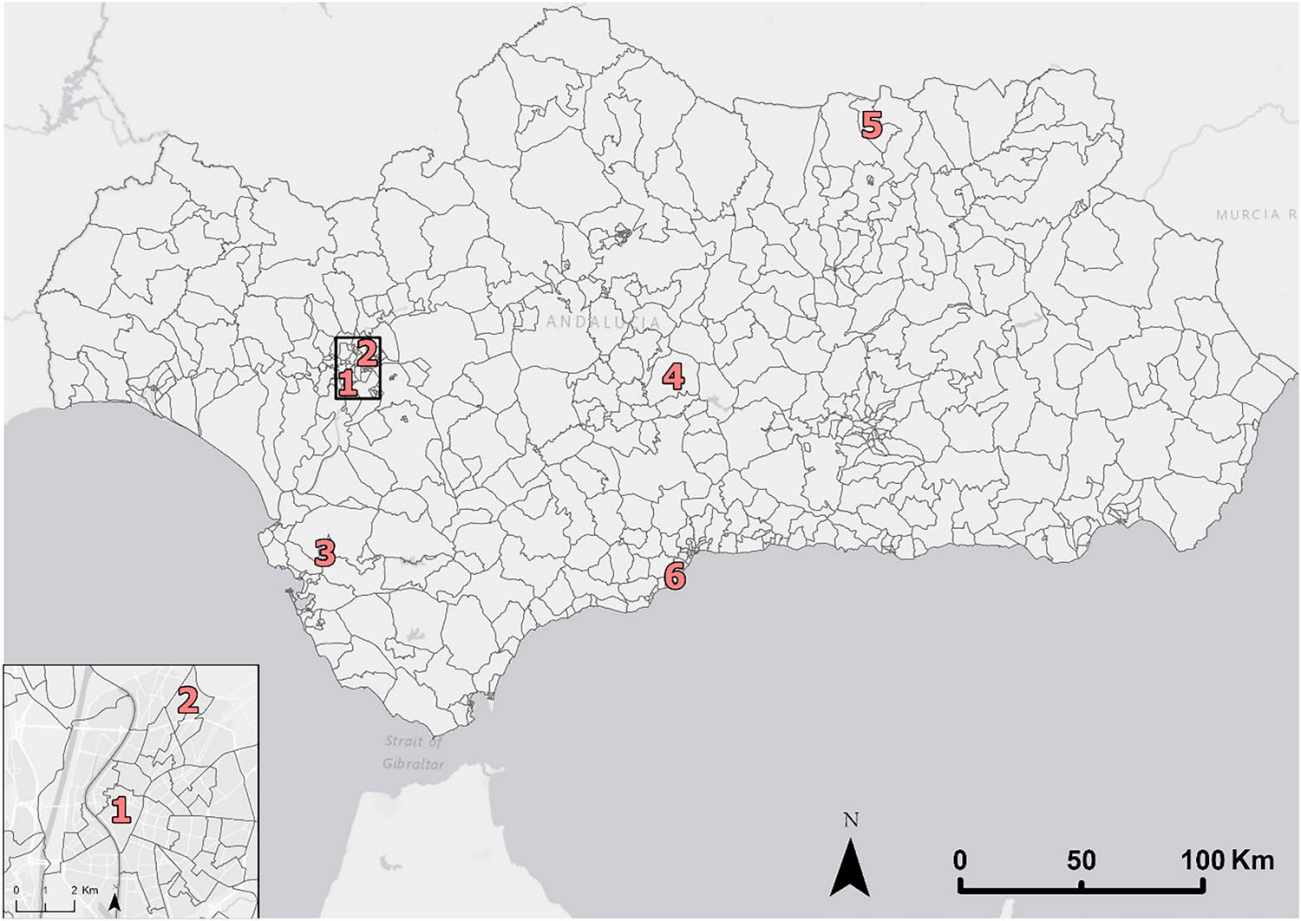
Spatial units whose estimated population has been analyzed and visualized in Fig. 7. Source: Own elaboration based on INE data from the *Estadística Experimental* portal. The program used to create the figure is *ArcGIS Pro* 3.2.1 GIS software. The figure points six spatial units that represent determined study cases [(1) touristic historical center from a major city, (2) residential neighborhood from a major city, (3) residential neighborhood from a big city, (4) middle-sized city located in the interior of the region, (5) small town located in the mountains, and (6) coastal touristic town] further analyzed and visualized in Fig. 7.

**Figure 7:**
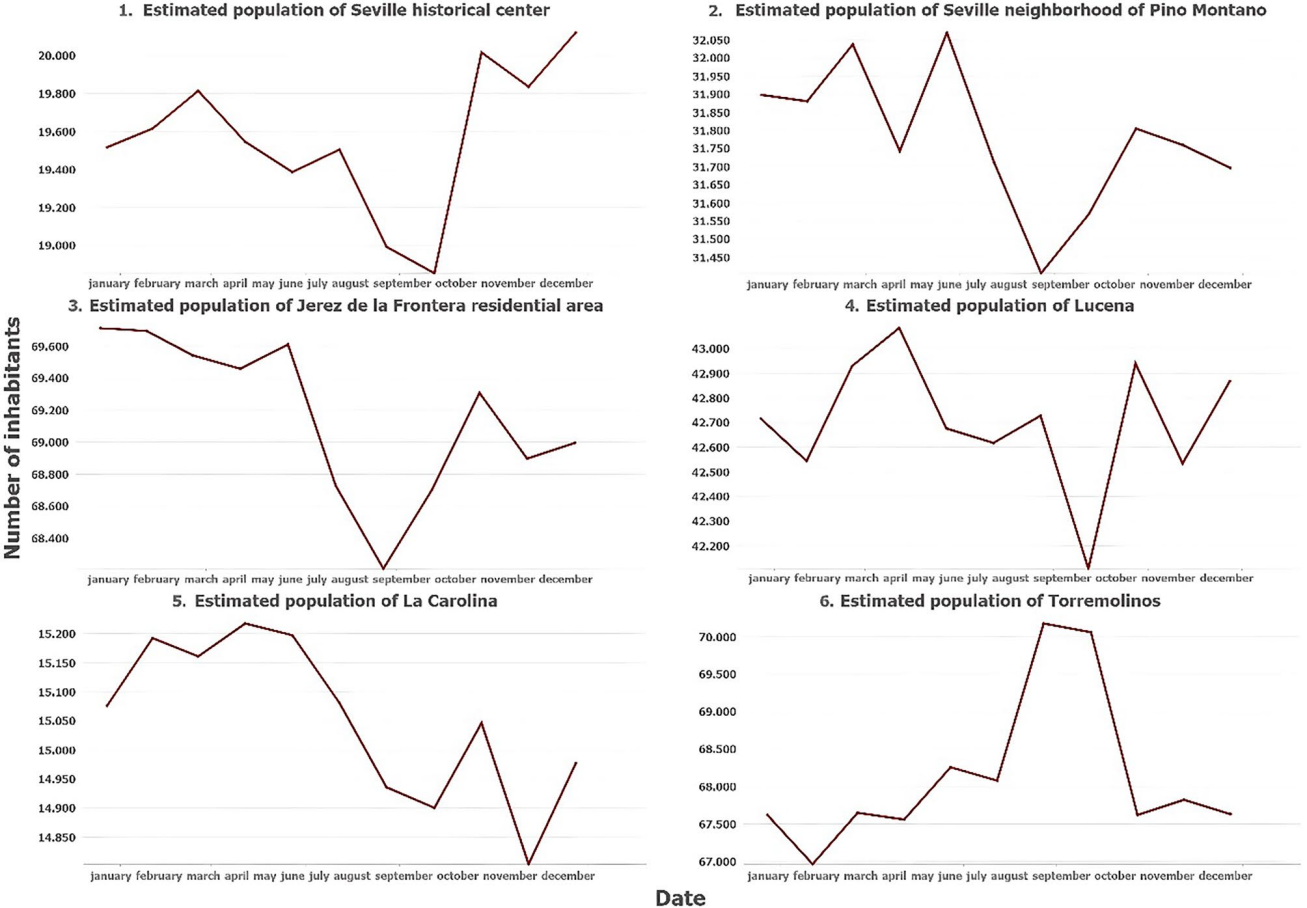
Examples of estimated population in various spatial units through the year 2021. Source: Own elaboration based on INE data from the *Estadística Experimental* portal. The program used to create the figure is *ArcGIS Pro* 3.2.1 GIS software. The figure shows graphs that indicate the estimated population obtained through the year in six different spatial units (a touristic historical center from a major city, a residential neighborhood from a major city, a residential neighborhood from a big city, a middle-sized city located in the interior of the region, a small town located in the mountains, and a coastal touristic town).

With the methodology used to calculate the dynamic population on a daily basis, the population graph of a city or neighborhood throughout the year can be analyzed. Next, the population of six spatial units with different cases will be analyzed (Fig. 6). For example, the historic center of Seville, an area with a very high concentration of tourism, presents stability throughout the first month’s year 2021, except for the month of August, where the strong heat makes tourism unsustainable. Then, the estimated population skyrockets in the fall season because the recovery of tourist and leisure mobility that had been reduced due to COVID-19 (Fig. 7, panel 1). If we analyze a neighborhood with a residential behavior in the same city (such as the Pino Montano neighborhood located to the north of the city of Seville), we observe in the first months of the year the estimated population was higher due to population remaining in their homes because of the mobility restrictions caused by COVID-19. Then after the end of the mobility restrictions there is prolonged decrease in the number of inhabitants, a situation that may be due to the displacement of residents to other neighborhoods of the city where employment and other services are located (Fig. 7, panel 2).

Most of the population units in Andalusia share several elements in common in their population graphs, whether in regional centers with strong population such as the historic center of Jerez de la Frontera (Fig. 7, panel 3), in medium-sized inland cities such as Lucena (Fig. 7, panel 4), or in small towns located in the mountains such as La Carolina (Fig. 7, panel 5). These elements consist of a higher population in the first months of the year (phenomenon that may be due to the recovery of the population after the COVID-19 pandemic and the reduction of mobility), a sharp decline in summer, and a progressive recovery in autumn (the population increases due to their return from vacation destinations, but at lower levels than at the beginning of the year because mobility increases as there are no restrictions caused by COVID-19). On the other hand, coastal cities such as Torremolinos follow an opposite trend because they are the main tourist destination of the territory during the summer period, having a significant floating population that they lack in the rest of the year (Fig. 7, panel 6).

**Figure 8:**
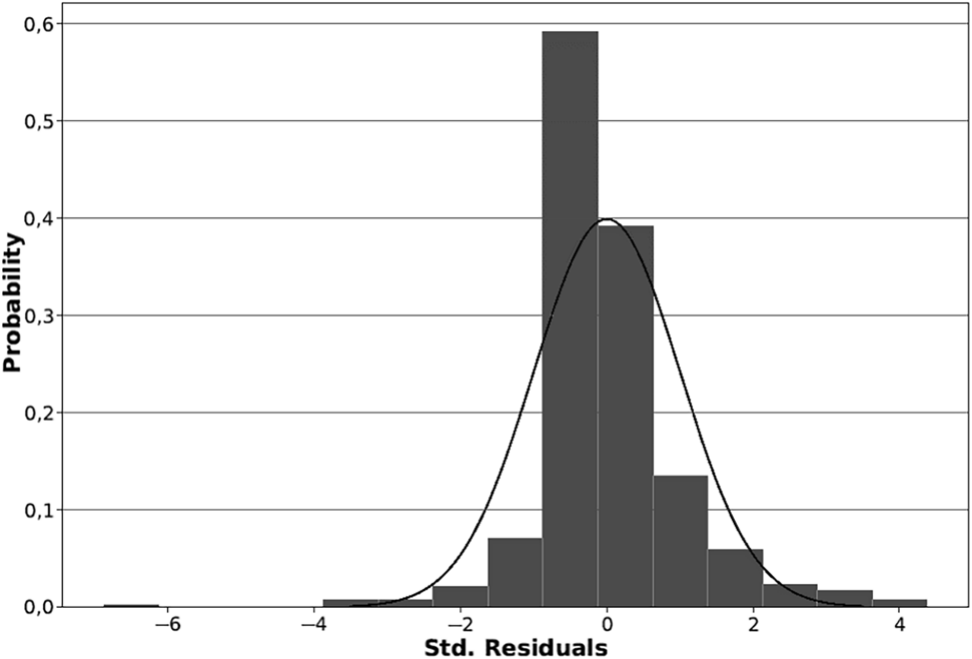
Histogram of standardized residuals in Andalusia spatial units between 2021 official population data from INE and the average obtained from all the population stock values of each spatial unit during the entire year. Source: Own elaboration based on INE data from the *Estadística Experimental* portal. The program used to create the figure is *ArcGIS Pro* 3.2.1 GIS software. The figure shows a histogram of the standard deviations obtained from an OLS model that used the two mentioned variables.

The results of the OLS regression model carried out between the official population data and the estimated population means for each special unit present an R2 value of 0.77, which indicates that the mobile phone data present a high fit and the results obtained are accurate for the most part. The distribution of the residuals between the official population data of the INE and the average of the estimated population values ​​throughout the year for each special unit show a normal and symmetrical distribution in the histogram (except for a single case that shows an enormous negative deviation) (Fig. 8).

**Figure 9:**
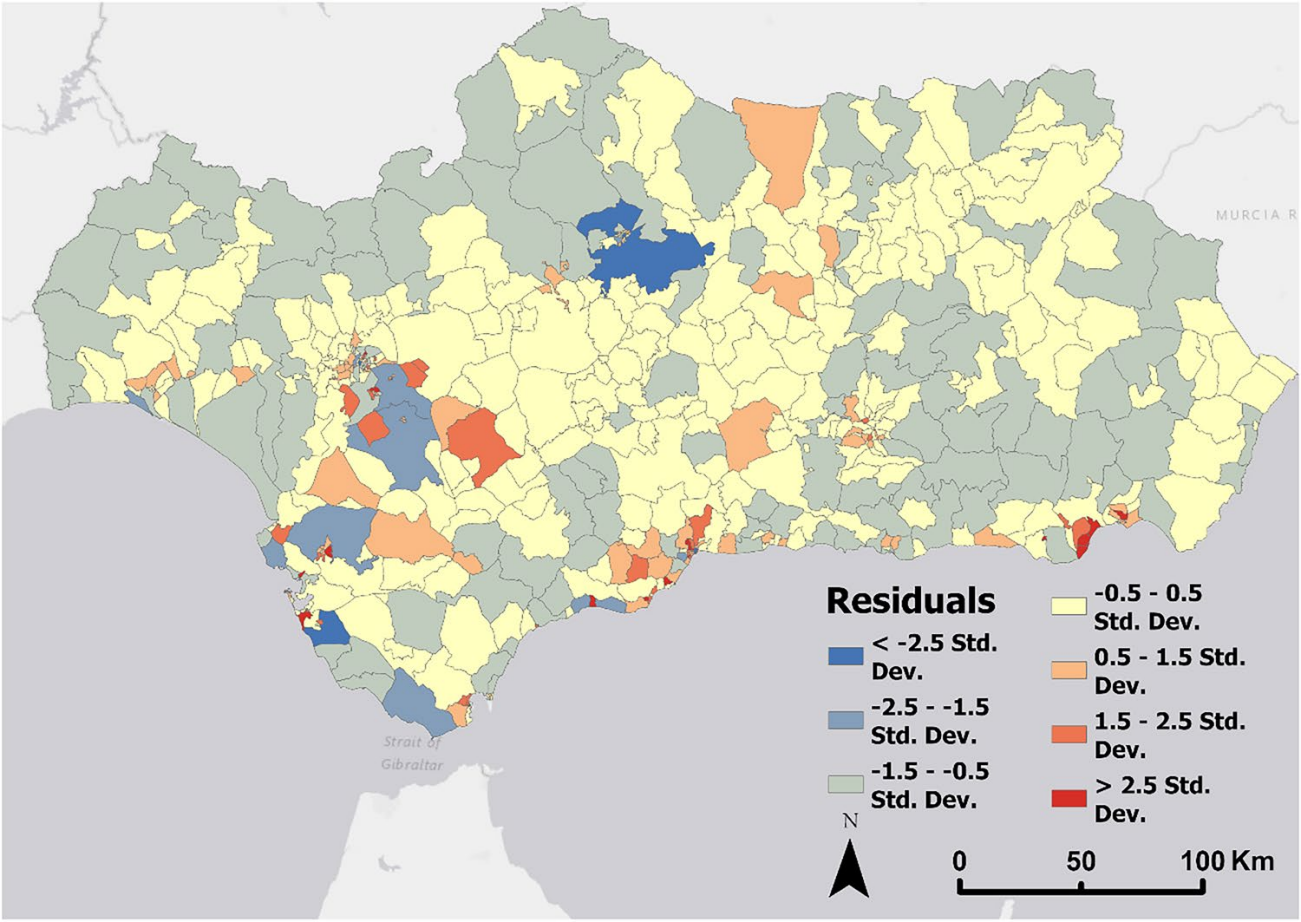
Standardized residuals in Andalusia spatial units between 2021 official population data from INE and the average obtained from all the population stock values of each spatial unit during the entire year. Source: Own elaboration based on INE data from the *Estadística Experimental* portal. The program used to create the figure is *ArcGIS Pro* 3.2.1 GIS software. The figure shows a choropleth representation of the standard deviations obtained from an OLS model that used the two mentioned variables.

The spatial unit with strange behavior, as can be visualized on Fig. 9, is the section of the municipality of Cordoba that does not correspond to the city itself, but to the countryside and the rural neighborhoods that surround it. Therefore, this spatial unit has a low population according to official data but a high population according to phone data. Another reason for this behavior may be due to the intense railway activity that the municipality of Cordoba presents and that generates noise: Cordoba is the main railway junction in Andalusia, where travelers commuting between Seville, Malaga, Granada and Jaen pass through. Furthermore, all trains traveling between any city in Andalusia and the Spanish capital of Madrid pass through Cordoba.

Moran's I test for spatial autocorrelation presents a value of 0.04 which suggests a weak spatial autocorrelation. However, the p-value of 0.008 and z-score of 2.63 indicate that the autocorrelation is statistically significant. These results indicate that although the spatial autocorrelation is weak, it is sufficiently consistent and not a product of chance. This slight but significant trend that similar values tend to be close to each other in space can be clearly visualized in the cartography of the distribution of the residuals.

Overall, the spatial distribution of residuals tends to be neutral in most of the region, with an overestimation of the estimated population from mobile phone data in the historic centers of the most populated cities in Andalusia and in mountain towns of the north and east of the region, and an underestimation of this estimated population in the residential neighborhoods and dormitory cities of the main metropolitan areas, and in the most densely populated coastal areas (especially the metropolitan areas of Cadiz, Malaga and Almería). In particular, the residential neighborhood located to the east of Jerez de la Frontera presents the highest positive residual value, having a high population but a low estimated population that may be due to the high displacement of the population to other spatial units that make up the city and to a low offer of employment, equipment and services (Fig. 9).

## Conclusions

This work has used data from OD matrices published by the INE throughout the year 2021 to visualize mobility flows and to estimate the daily population of each of the population units that make up the region of Andalusia over the year 2021 with the aim of studying how the recovery of mobility after the restriction periods caused by COVID-19 affects the different population flows between cities and the demographics of cities with different cases. These data correspond to the number of mobile phones registered in each mobility flow on a given day, which have subsequently been transformed into population values to mitigate the sociodemographic bias of these data. A methodology has been designed to first calculate the mobile phone population of a unit in a day, and then use a correction factor for the percentage of mobile phone use according to official data to transform this sample into a population stock. Finally, the population of each day has been estimated taking into account the full time period of one year. For this purpose, an interpolation formula has been established using the census population as adjustment data.

While traditional cartography shows limitations when it comes to representing mobility flows and changes in the population over long periods of time (for example, Figs. 2 and 4 showed only eight key days in a static way), web cartography allows these changes to be shown interactively and clearly during the entire time period. For that purpose, a web map has also been created, making it possible to display a greater amount of information thanks to the popups that indicate the name of the population unit and show more detailed information.

The results show how demographic changes in Andalusia are directly linked to the recovery of the population after the COVID-19 pandemic and the mobility between the place of residence and the place of work. The lower number of mobility flows in the first months of the year 2021, a period corresponding to the second alarm state of COVID-19, have shown more neutral demographic changes. In contrast, the recovery of mobility throughout the last 4 months of the year translates into stronger population changes.

The analysis of mobility flows corroborates these results, showing a direct relationship between the number of travelers detected in the data, the end of mobility restrictions caused by COVID-19, and the length of travel flows. Since mobility in the first months of the year is basically limited to trips between home and work, there are fewer travels and trips are concentrated in short-distance trips. After the end of mobility restrictions caused by COVID-19, both the number and length of trips increases. As mobile phone data were obtained mainly for home-work trips, the sample size is concentrated on trips between different areas of a city or between different cities in the same metropolitan area. Long mobility flows are lower and tend to correspond to tourist mobility between the interior and the coast or between inland cities with a high tourist impact such as Seville or Granada.

Based on the use of mobile phones during trips between home and work, it has been possible to draw two demographic profiles in the territory. The first profile shows an increase in population in the main cities of the region and a demographic decrease in a large part of the territory during working days, while during weekends the opposite phenomenon is experienced. The second profile occurs in summer, with an increase in population in most of Andalusia (especially on the coast) during working days, while large inland cities such as Seville and Cordoba suffer a sharp demographic decline.

The validation of the results obtained with respect to official population data using an OLS regression shows that mobile telephone data are an optimal source of data for demographic studies, both general and related to diseases or pandemics. Thanks to their high spatial and temporal detail, mobile phone data is useful to create synthetic and continuous populations. However, we must take into account the presence of noise that affects these results, such as the presence of infrastructure, equipment, services and job offers, or the degree of accessibility to a public transport system or a road network.

Mobile phone data tend to have limitations regarding accessibility, but it is a situation that is changing in recent years, because an increasing number of institutions and organizations realize the potential of this data, and share it so that it can be processed and investigated by the public. While other data sources such as Google focus on studying the mobility of citizens towards different types of establishments and aggregate the results by region, mobile phone data shows the mobility relationships between the different population units that make up the region, being a more effective data source to analyze metropolitan mobility. On the other hand, the lack of thematic information shows a need to complement the data obtained with other sources that include information about the type of places to which the population moves, such as precisely Google mobility data.

Although the methodology used in this work has sought to mitigate the effects of the biases presented by the use of mobile phones on the population (lower use of mobile phones by the elderly or children, population groups with a low socioeconomic level, and population living in rural areas), the use of these data continues to have a series of limitations without which it would be possible to delve deeper into the demographic changes of the territory. The data used lacked demographic or socioeconomic information, so that studies aimed at certain population groups cannot be compared. In addition, other information that could be of interest, such as details of trips or transport modes, is not available. The available data corresponded to a full day, so there is no information on mobility or demographic changes at different times of the same day, and therefore it is not possible to generate a more detailed footprint of the behavior patterns of the population in aspects related to commuting to the workplace or to other spaces. Finally, these data have been generated every three days, so that, although information is available showing the mobility and population of the territory on a working day and a weekend day, it does not take into account the differences that may exist in the behavior of society on different working days of the same week.

INE has noticed all these limitations and starting 2022 they have begun to publish daily mobility data from mobile phones that includes more detailed information such as the reason for the trip, the gender of the traveler, the transport mode, or their socioeconomic information. However, these data are not available for the time period analyzed in this work, although it remains a future line of research and an evolution of the present work. Another limitation to take into account precisely from this addition of new data lies in its anonymization and the protection of the privacy of mobile phone users.

This work presents several relevant lines of research for the future. The main one is the potential to calculate the dynamic population of a population in different time periods of the same day (although, as previously mentioned, it depends on the institutions publishing data with a higher level of temporal detail). Another line of research consists of identifying the main land use in each population unit according to cadastral data, with the goal of identifying the main work and residential locations of the cities, and spaces oriented to other uses such as culture or tourism.

## Data Availability

Mobile phone data used for this work is openly available in the *Estadistica Experimental* portal by Instituto Nacional de Estadística in the data download section of the following url: https://www.ine.es/experimental/movilidad/experimental_em4.htm. The web map HTML document (named as index) and the estimated population data geojson files are stored in the following repository: https://osf.io/c2yz4/.
